# Human Amniotic Mesenchymal Stem Cells Alleviate aGVHD after allo-HSCT by Regulating Interactions between Gut Microbiota and Intestinal Immunity

**DOI:** 10.1007/s12015-023-10522-4

**Published:** 2023-03-04

**Authors:** Xiaoyin Bu, Junhui Wang, Zhao Yin, Weifeng Pan, Liping Liu, Hua Jin, Qifa Liu, Lei Zheng, Haitao Sun, Ya Gao, Baohong Ping

**Affiliations:** 1 Department of Hematology, Nanfang Hospital, Southern Medical University, Guangzhou, 510515 China; 2 Department of Hematology, Huiqiao Medical Center, Nanfang Hospital, Southern Medical University, Guangzhou, 510515 China; 3 Department of Laboratory Medicine, Nanfang Hospital, Southern Medical University, Guangzhou, 510515 China; 4 Department of Laboratory Medicine Clinical Biobank Center, Microbiome Medicine Center, Zhujiang Hospital, Southern Medical University, Guangzhou, 510280 China

**Keywords:** Acute graft-versus-host disease, Amniotic mesenchymal stem cells, Gut microbiota, Intestinal barrier, Intestinal immunity

## Abstract

Acute graft-versus-host disease (aGVHD) after allogeneic hematopoietic stem cell transplantation poses one of the most vexing challenges. Gut microbiota dysbiosis can proceed aGVHD and mesenchymal stem cells (MSCs) have promising therapeutic potential for aGVHD. However, whether hAMSCs affect the gut microbiota during aGVHD mitigation remains unknown. Accordingly, we sought to define the effects and underlying mechanisms of human amniotic membrane-derived MSCs (hAMSCs) regulating the gut microbiota and intestinal immunity in aGVHD. By establishing humanized aGVHD mouse models and hAMSCs treatment, we found that hAMSCs significantly ameliorated aGVHD symptoms, reversed the immune imbalance of T cell subsets and cytokines, and restored intestinal barrier. Moreover, the diversity and composition of gut microbiota were improved upon treatment with hAMSCs. Spearman’s correlation analysis showed that there was a correlation between the gut microbiota and tight junction proteins, immune cells as well as cytokines. Our research suggested that hAMSCs alleviated aGVHD by promoting gut microbiota normalization and regulating the interactions between the gut microbiota and intestinal barrier, immunity.

**Graphical Abstract:**

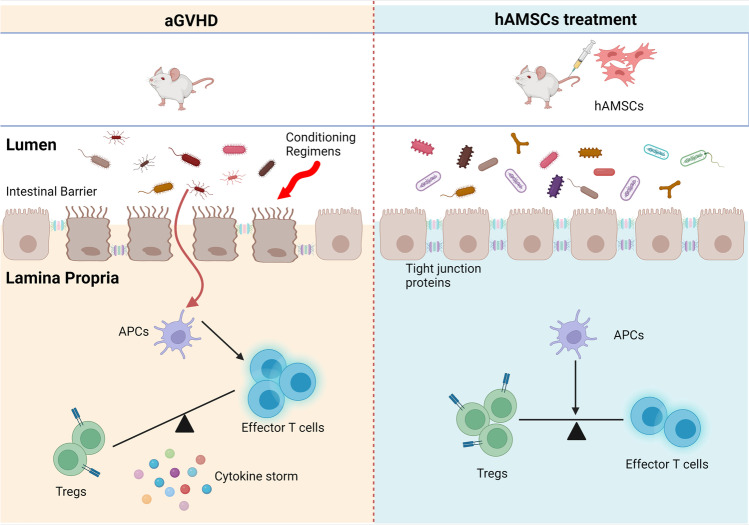

## Introduction

The primary advantage of allogeneic hematopoietic stem cell transplantation (allo-HSCT) is the potent anti-tumor response due to a graft-versus-leukemia (GVL) effect which is triggered by the recognition of peptides arising from differences in genomic polymorphisms between patient and donor. However, acute graft-versus-host disease (aGVHD) revolves around the development of recipient tissue damage which due to the attack by the allo-reactive donor T cells, and represents a life-threatening major complication in allo-HSCT [1, 2].

Gut microbial disorder has been reported to induce inflammatory responses and correlate with inflammatory associated diseases [3, 4]. Numerous studies have indicated that perturbations in the gut microbiota are an essential factor triggering aGVHD after allo-HSCT, and become a novel target for treatment [5, 6]. Specifically, gut microbiota dysbiosis in aGVHD is usually characterized by the loss of intestinal bacterial diversity and outgrowth of opportunistic pathogens [6]. In gastrointestinal GVHD, the compromised mucosal barrier initiates activation of host antigen-presenting cells and donor T cell culminating in T-cell differentiation along pathogenic type-1 and type-17 paradigms at the expense of tolerogenic regulatory T-cell patterns [7]. Primarily on the basis of 16S ribosomal RNA (16S rRNA) sequencing indicated that multiple species of gut microbiota have been associated with GVHD, and for example, butyrate-producing clostridia are associated with the maintenance of epithelial barrier function and attenuation of acute GVHD [8].

Mesenchymal stem cells (MSCs) have gained vast attention in the past decade due to their self-renewal and multilineage differentiation potentials, as well as immunomodulatory properties [9]. MSCs are thought to enable damaged tissues from balanced inflammatory and regenerative microenvironment in the presence of vigorous inflammation and have been widely used to treat immune-based disorders, in which the most successfully clinical application is involved in GVHD treatment [10]. Human amniotic mesenchymal stem cells (hAMSCs) are one of the perinatal stem cells which possess embryonic stem cell-like differentiation capability and adult stem cell-like immunomodulatory properties. Sharing phenotypes similar to typical MSCs, hAMSCs have become a promising source of stem cells owing to the ease, noninvasive and safety of tissue acquisition, abundant cell yield, very low ethical and moral disputes compared with stem cells from other sources. Human AMSCs have the advantages of low immunogenicity and no tumorigenicity, making them the ideal cell source for cell therapy [11, 12]. In vitro experiments have confirmed that hAMSCs displayed a higher proliferative capacity and greater long-term growth ability than bone marrow-derived MSCs [13, 14]. Numerous studies have demonstrated that hAMSCs hold the potential to ameliorate many inflammatory diseases, including inflammatory bowel disease, osteoarthritis and autoimmune disease [11]. Our recent study has shown that hAMSCs ameliorated aGVHD through regulating the balance of T effector and Treg cells [15]. However, the effect and mechanism of hAMSCs on gut microbial disorder in GVHD are unknown. Here, we aim to investigate the potential impact of hAMSCs on aGVHD as well as gut microecosystem. It would be the first study to reveal the gut microbiota and its correlation with the intestinal immunity following hAMSCs treatment of aGVHD.

## Materials and Methods

### Isolation, Culture and Identification of hAMSCs

The hAMSCs were extracted as previous described [15]. Third-passage (P3) cells were harvested from phenotype identification through staining with antibodies against CD34 (581, BD Pharmingen, USA), CD45 (HI30, BD Pharmingen, USA), HLA-DR (G46-6, BD Pharmingen, USA), CD11b (ICRF44, BD Pharmingen, USA), CD90 (5E10, BD Pharmingen, USA), CD73 (AD2, BD Pharmingen, USA) and CD105 (SN6, ebioscience, USA), and then analyzed by flow cytometer (BD FACS Canto II). The cells were checked for their multilineage differentiation using a specific induction medium (BGscience, China), and stained with Oil Red-O and Alizarin Red for adipogenesis and osteogenic differentiation, respectively. hAMSCs at passages 3 to 6 were used for the experiments.

### Human PBMCs Collection

Plasma samples were procured from healthy volunteers with written informed consent. Human peripheral blood mononuclear cells (PBMCs) were isolated from peripheral blood by Ficoll-Hypaque (Tianjin Haoyang, China) density centrifugation, washed with PBS, suspended in red blood lysis buffer (Solarbio, China) at 4 °C for 15 min. Wash again and then suspended in PBS for tail vein injection into NPG mice.

### Mice

8–10 weeks of age and 25–30 g male and female NPG mice were purchased from Beijing Vitalstar Biotechnology Co., Ltd. and reared under SPF conditions on a 12 h light–dark cycle with constant temperature and humidity. All animal procedures were approved by the Southern Medical University Institutional Animal Care and Use Committee (No. L2019132) in accordance with the National Health and Medical Research Council of China Guidelines on Animal Experimentation.

### Acute GVHD Animal Model and Treatment

The aGVHD mouse model were established as previous described [15]. For treatment, 5 × 10^5^ hAMSCs per mouse (herein hAMSCs group) and PBS (herein aGVHD group) were injected via tail vein into each mouse on the third day after transplantation. In all mouse models, mice were checked every two days for morbidity and weight changes. Each mouse was scored for pathological features including weight loss, hunched posture, ruffled fur, skin lesions, reduced mobility and diarrhea and then graded according to the aGVHD clinical scoring system adapted from the one originally described by Cooke [16]. In some cases, mice in aGVHD group suffered from severe aGVHD and the mice in hAMSCs group on the third day after treatment were sacrificed to harvest the peripheral blood and target organs including livers, spleens, lungs and intestines. The feces of these mice were collected before sacrifice.

### GFP-labeled hAMSCs and in vivo Tracing

GFP-labeled hAMSCs were established and identified as previous described [15]. In brief, we transfected green fluorescence protein (GFP) gene into hAMSCs with lentivirus as vector. 5 × 10^5^ GFP-labeled hAMSCs suspended in 500 μL PBS were injected into aGVHD mice via the tail vein. After 24 h and 72 h, the mice were euthanized to harvest the intestines and then generated frozen sections. DAPI counterstaining was used to distinguish the recipient cells.

### Histopathological Evaluation

The aGVHD target organs were collected at the time of necropsy, and then processed to paraffin-embedded blocks to generate 5-μm-thick sections for hematoxylin and eosin staining (H&E). Histological scores were assessed based on the tissues structure destruction and lymphocyte infiltration [17]. For immunohistochemistry, sections were deparaffinized, rehydrated and treated with 3% H_2_O_2_ in methanol for 20 min to inactivate endogenous peroxidase activity. Then sections were subjected to antigen retrieval and treated with serum albumin for 1 h. Subsequently, the sections were incubated with rabbit anti-human CD45 antibodies (EP322Y, Abcam, England) or ZO-1 antibodies ((EPR19945-296, Abcam, England) for 1 h at 37 °C. The bound antibodies were detected with HRP-conjugated secondary antibody (Genetech, USA) and visualized with DAB. The positive area was analyzed by Image J.

### 16S rRNA Sequencing of Fecal Microbiota

Fecal genomic DNA was extracted from 0.1 g frozen fecal samples using an E.Z.N.A.® stool DNA Kit (Omega Bio-Tek, Norcross, GA, U.S.) following the manufacturer's protocol. Using genomic DNA as the template, specific primers (338F: 5′-ACTCCTACGGGAGGCAGCA-3′, and 806R: 5′-GGACTACHVGGGTWTCTAAT-3′) with barcodes and PremixTaq (TaKaRa, China) were used to amplify the V3-V4 hypervariable regions of the bacterial 16S rRNA gene. After amplification and purification, the whole genome of the sample was sequenced on the Illumina Hiseq or Miseq high-throughput sequencing platform by Magigene Technology Co., Ltd. (Guangzhou, China) to obtain raw data in FASTQ format. Sequence data analyses were mainly performed using Magichand platform.

### Flow Cytometric Analysis

The aGVHD target organs and peripheral blood of mice were obtained and prepared into single cell suspension. Cells were stained with monoclonal antibodies against CD3 (UCHT1, Biolegend, USA), CD4 (RPA-T4, Invitrogen, USA), CD8 (RPA-T8, Biolegend, USA), CD25 (BC96, Biolegend, USA) or isotype matched control IgG (eBioscience, USA) for 30 min at room temperature in the dark. Then the cells were processed with Lysing Buffer (BD Pharm Lyse™) at 4 °C in the dark for 15 min. Intracellular Foxp3 (236A/E7, Invitrogen, USA) staining was performed according to the manufacturer’s recommendations (Fixation/Permeabilization Solution Kit; eBioscience). Then polychromatic flow cytometric analyses were performed on Flow Cytometer.

### Cytokines Quantitation

The levels of cytokines in the serum and tissue supernatant of mice were determined using the Cytometric Bead Array (CBA) Human Th1/Th2/Th17 cytokine kit (BD Pharmingen, USA) according to the manufacturer’s instructions. Briefly, 50 μL of each of the supernatant samples was incubated with 50 μL of the mixed human Th1/Th2/Th17 cytokine capture beads and 50 μL of the human Th1/Th2/Th17 PE detection reagent at room temperature in the dark, then suspended for flow cytometry analysis 3 h later.

### Quantitative Real-time PCR

Total RNA was extracted from mouse colonic tissue after isolation using Trizol Reagent (TaKaRa, China). Total RNA was used as a template to reverse transcribe cDNA. β-actin was used as an internal reference. The mRNA expression was measured by qPCR by using the following primers: ZO-1-F, 5’-ACCACCAACCCGAGAAGAC-3’, and ZO-1-R, 5’-CAGGAGTCATGGACGCACA-3’; Occludin-F, 5’-TTGAAAGTCCACCTCCTTACAGA-3’, and Occludin-R, 5’-CCGGATAAAAAGAGTACGCTGG-3’; β-actin-F, 5’-GGCTGTATTCCCCTCCATCG-3’, and β-actin-R, 5’-CCAGTTGGTAACAATGCCATGT-3’. The 2^−△△Ct^ method was used to measure the RNA expression levels.

### Enzyme-Linked Immunosorbent Assay (ELISA)

The levels of D-LA and DAO in plasmas were measured using ELISA kits (Jingmei, China) according to the manufacturer’s instructions. Briefly, the collected plasma supernatants were put into 96-well specific antibody-coated plates, respectively. Absorbance was recorded at 450 nm using a microplate reader (Multiskan MK3, Thermo Fisher Scientific).

### Statistical Analysis

Data were presented as mean ± SD. Independent samples *t*-test was performed and visualized using GraphPad Prism8.0. 16S rRNA sequencing data analyses were performed using Magichand platform (http://cloud.magigene.com/). Correlation analysis between gut microbiota and immunity was conducted by R packages (v3.6.3) to display correlation matrix. P values < 0.05 were considered statistically significant. * *p* < 0.05, ** *p* < 0.01, *** *p* < 0.001, **** *p* < 0.0001, n.s. not significant.

## Results

### Identification of hAMSCs

**Fig. 1:**
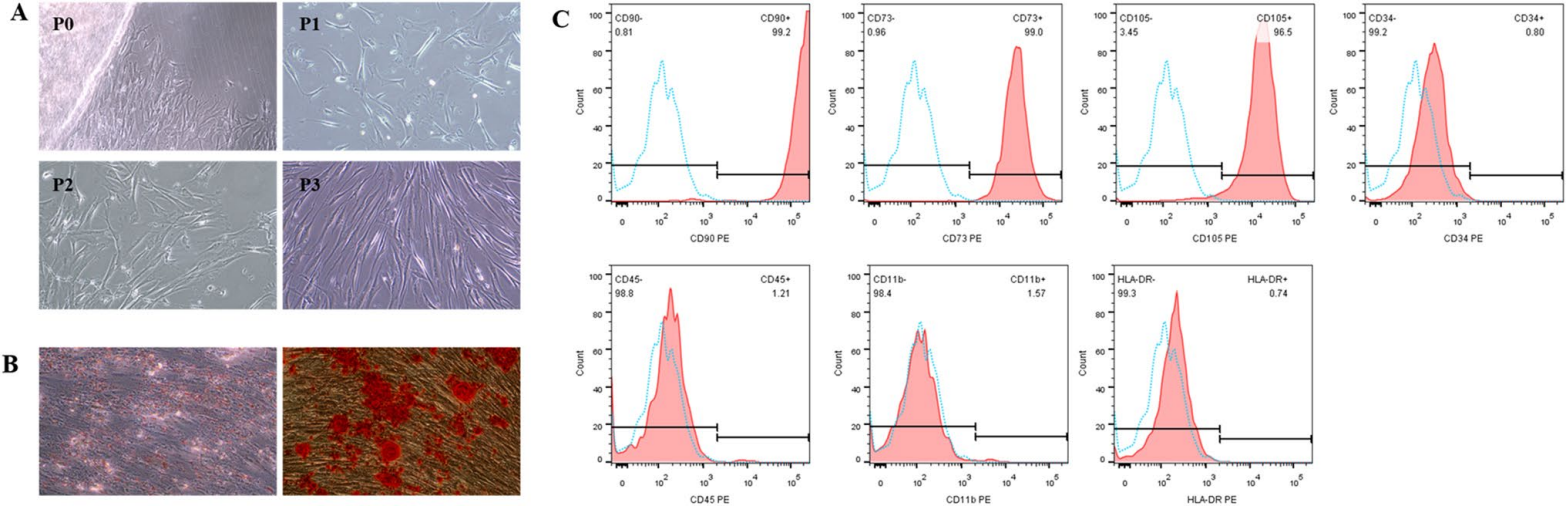
Phenotypic and functional characterization of hAMSCs. (**A**) Representative micrographs of hAMSCs from passage 0 to 3 (no staining, 100 ×). (**B**) Multilineage differentiation ability of hAMSCs. Adipogenic differentiation of hAMSCs (left) were stained with Oil Red-O, and osteoblastic differentiation of hAMSCs (right) were stained with Alizarin Red (staining, 100 ×). (**C**) Flow cytometric analysis indicating hAMSCs positive for CD90, CD105 and CD73 while negative for CD34, CD45, CD11b and HLA-DR

The hAMSCs exhibited polygon or fibroblast-like morphology in culture (Fig. 1A) and were strongly positive for surface expression of MSC specific markers CD90, CD105 and CD73, but negative for CD34, CD45, HLA-DR and CD11b (Fig. 1C). As for multilineage differentiation, our results showed that hAMSCs demonstrated adipocytic and osteoblastic differentiation (Fig. 1B).

### hAMSCs Significantly Alleviated aGVHD Symptoms in Mice

**Fig. 2:**
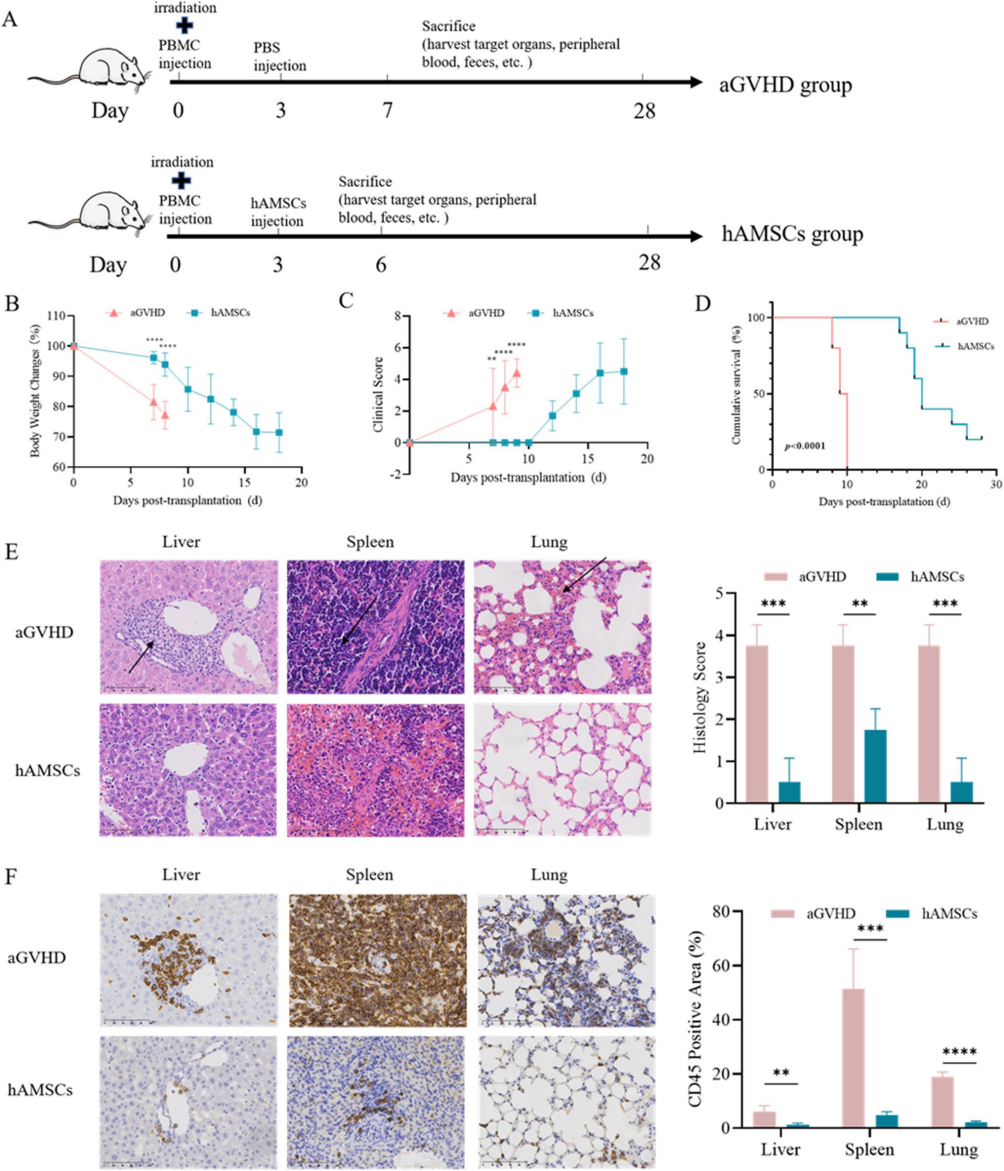
hAMSCs alleviated aGVHD in NPG mice. (**A**) A schematic diagram illustrating administration schedule of hAMSCs in aGVHD mouse models. (**B**) Changes of body weight in different groups (*n* = 10). (**C**) aGVHD clinical score (based on weight loss, hunched posture, ruffled fur, skin lesions, reduced mobility and diarrhea) in different groups (*n* = 10). (**D**) Survival among different groups (*n* = 10). (**E**) Representative microscopic pictures of H&E staining (400 ×) and histology score (based on the tissues structure destruction and lymphocyte infiltration) (*n* = 4). (**F**) Representative microscopic pictures of immunohistochemistry (400 ×) and the proportion of CD45 positive area (*n* = 4). Values were presented as mean ± SD

We firstly evaluated the therapeutic effect of hAMSCs on aGVHD. Compared with aGVHD group, hAMSCs significantly ameliorated aGVHD, as evidenced by the markedly reduced weight loss (Fig. 2B) and decreased aGVHD clinical score (Fig. 2C). Furthermore, hAMSCs group had a significantly higher survival rate when compared to aGVHD mice (Fig. 2D). The pathological histology analysis showed that aGVHD caused severe tissue damage and leukocyte infiltration. hAMSCs, as demonstrated by H&E, attenuated tissue disruption and infiltration of exogenous T cells in aGVHD target organs (Fig. 2E). Consistently, the results of IHC also confirmed that hAMSCs improved abnormal pathological manifestations (Fig. 2F). These results suggested that hAMSCs had the potential to ameliorate aGVHD symptoms in aGVHD mice.

### hAMSCs Affected the Host Immunity in aGVHD Mice

**Fig. 3:**
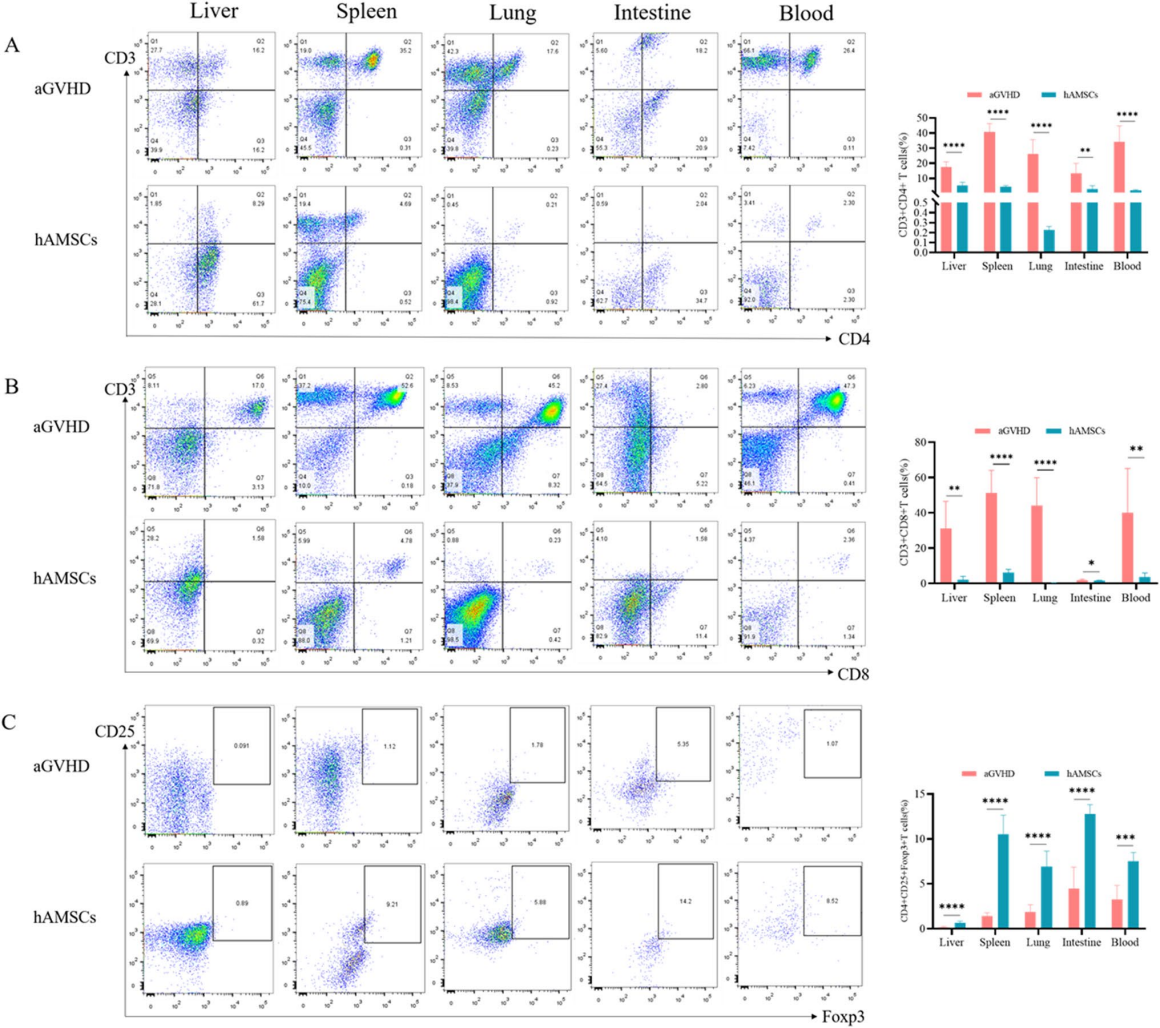
hAMSCs inhibited donor T cell expansion while enhanced Tregs generation or expansion in vivo. (**A**) Flow cytometry analysis of CD3 + CD4 + T cells(*n* = 6), (**B**) Flow cytometry analysis of CD3 + CD8 + T cells(*n* = 6), (**C**) Flow cytometry analysis of CD4 + CD25 + Foxp3 + Tregs (*n* = 6). Values were presented as mean ± SD

**Fig. 4:**
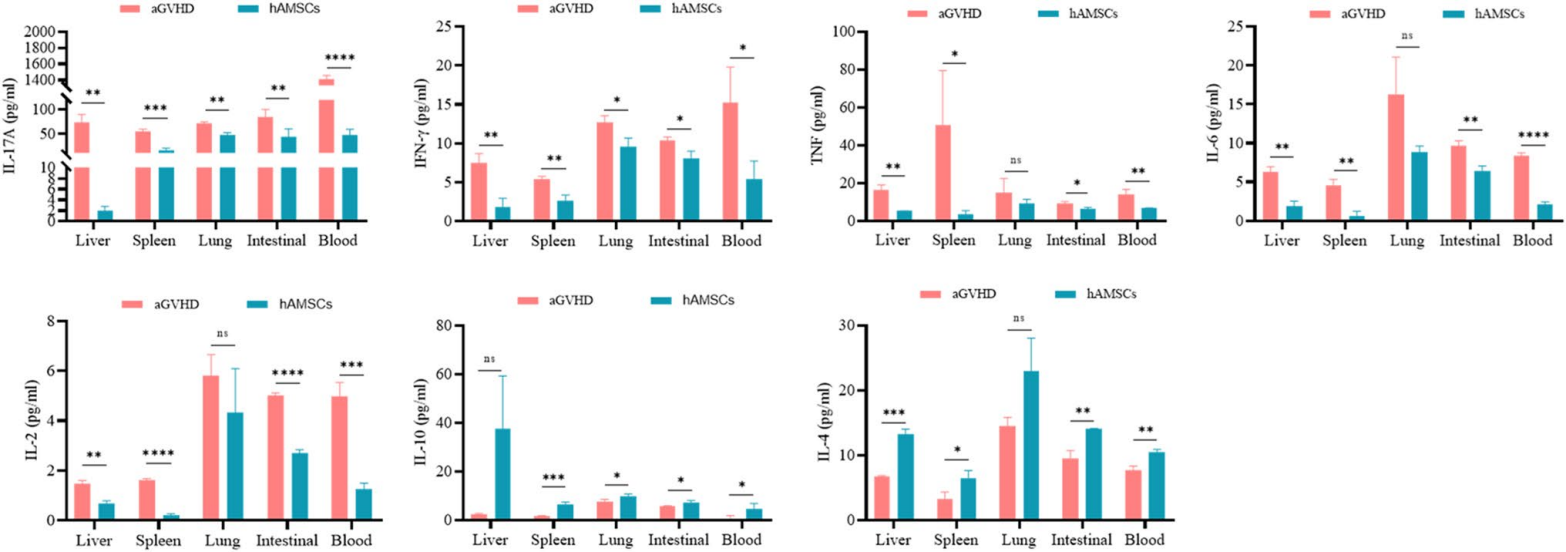
hAMSCs downregulated pro-inflammatory cytokines IL-17A, IFN-γ, TNF, IL-6, IL-2 and upregulated anti-inflammatory cytokines IL-10 and IL-4 in vivo (*n* = 5). Values were presented as mean ± SD

To understand the underlying immunomodulatory mechanisms by which hAMSCs reduce aGVHD, we measured the activation and expansion of T cells in peripheral blood and target organs. Flow cytometry assay revealed that hAMSCs remarkably inhibited the number of CD3 + CD4 + T and CD3 + CD8 + T cells in the blood and target organs (Fig. 3A, B). Moreover, the proportion of CD4 + CD25 + Foxp3 + Tregs in the blood and target organs of mice in hAMSCs group was significantly higher than that in aGVHD group (Fig. 3C). We next investigated the effect of hAMSCs on the cytokine profile (Fig. 4). hAMSCs administration resulted in an obviously decrease in the IL-17A, IFN-γ, TNF, IL-6 and IL-2 levels in the target organs and blood compared to those of aGVHD group, except for TNF level in the spleen. At the meantime, in addition to IL-10 level in the liver, hAMSCs treatment mice displayed substantially increased levels of IL-10 and IL-4 in the target organs and blood. Taken together, hAMSCs played a protective role in aGVHD through reversing the immune imbalance of T cell subsets and cytokine storm.

### hAMSCs Ameliorated Intestinal Barrier Damage in aGVHD Mice

**Fig. 5:**
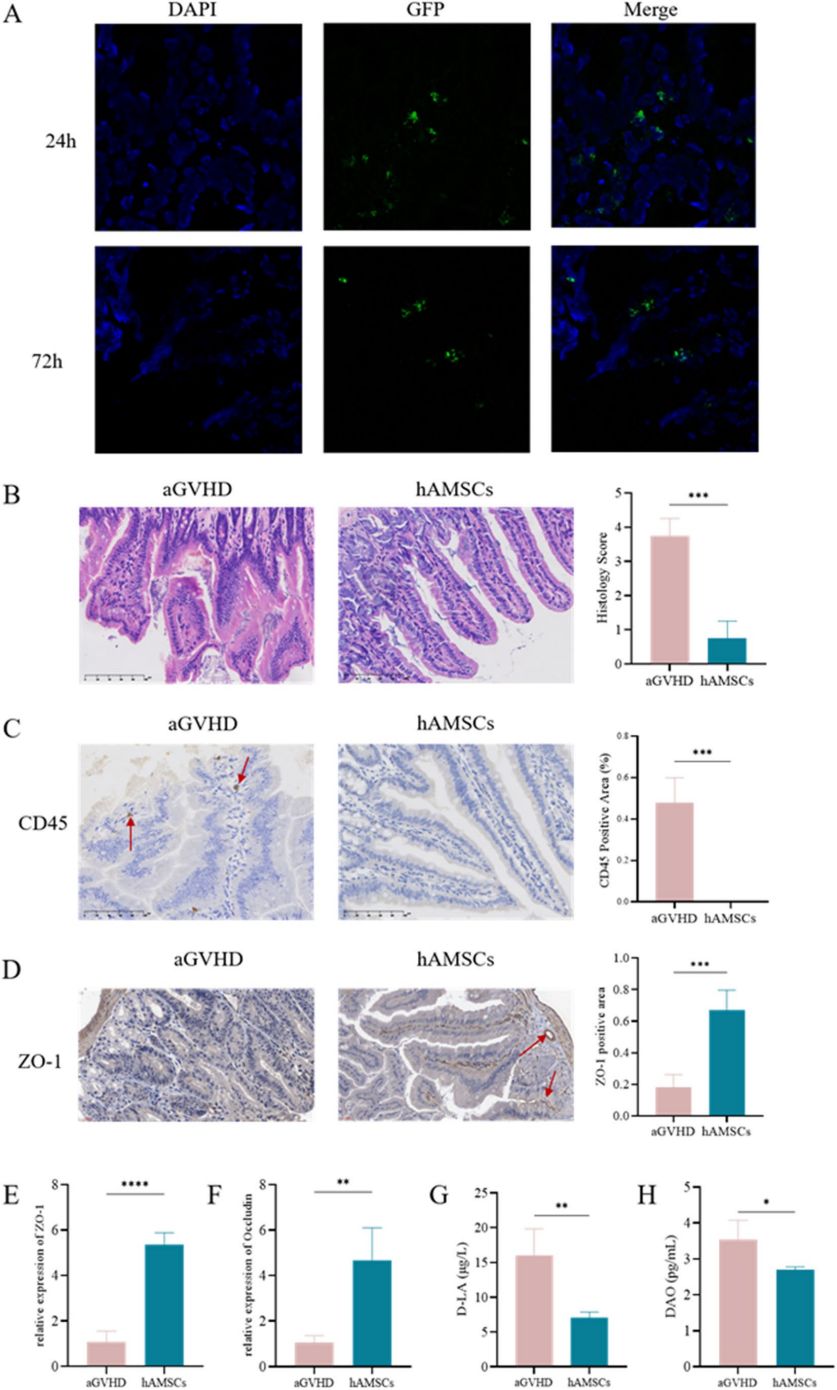
hAMSCs ameliorated intestinal barrier dysfunction in aGVHD mice. (**A**) GFP-labeled hAMSCs infiltrated into the intestines. (**B**) Representative microscopic pictures of H&E staining of intestines (400 ×) and histology score (based on the tissues structure destruction and lymphocyte infiltration) (*n* = 4). (**C**) Representative microscopic pictures of immunohistochemistry (400 ×) of intestines and the proportion of CD45 positive area (*n* = 4). (**D**) Representative microscopic pictures of immunohistochemistry (400 ×) of intestines and the proportion of ZO-1 positive area (*n* = 4). (**E**) mRNA expression levels of ZO-1 and Occludin (*n* = 3). (**F**) Plasma levels of D-LA and DAO (*n* = 4). Values were presented as mean ± SD

To investigate whether hAMSCs could repair intestinal barrier in aGVHD, we firstly observed whether hAMSCs could migrate to the intestines. As shown in Fig. 5A, GFP-labeled hAMSCs were detected in the intestines after injection into mice after 24 h and 72 h, demonstrating that transplanted hAMSCs could engraft and infiltrate in the intestines. Then we designed histopathological evaluation of small intestinal tissue. In aGVHD group, the intestine was eroded and necrotic, the intestinal villi were obviously destroyed, the villi were broken and massive leukocyte infiltrated in the intestinal mucosa, submucosa and lamina propria. The intestinal villi were regularly arranged, and there was no intestinal villus breakage or detachment of the villous epithelium after hAMSCs treatment (Fig. 5B). Human lymphocyte infiltration was also observed by immunohistochemistry in the intestines in aGVHD group, whereas hAMSCs treatment could reduce their infiltration (Fig. 5C). To assess the protective effect of hAMSCs on intestinal mucosal integrity, we performed immunohistochemistry to detect the effect on ZO-1. In hAMSCs treatment group, the expression level of the tight junction protein ZO-1 was significantly increased compared with aGVHD group (Fig. 5D). We also examined the mRNA expression levels of intestinal tight junction proteins (TJs) and plasma levels of D-lactic acid (D-LA) and diamine oxidase (DAO) to determine intestinal barrier function and intestinal permeability. When administrated intravenously, hMASCs could significantly increase the mRNA expression levels of ZO-1 and Occludin, the key proteins of TJs (Fig. 5E, F), while decrease the levels of D-LA and DAO (Fig. 5G, H). Hence, when aGVHD occurred, the intestinal barrier was damaged with intestinal tight junction proteins reduction and the intestinal permeability increasing. Meanwhile, hAMCSs could markedly reversed the disruption of the intestinal barrier.

### Gut Microbiota Alternations After hAMSCs Treatment in aGVHD Mice

**Fig. 6:**
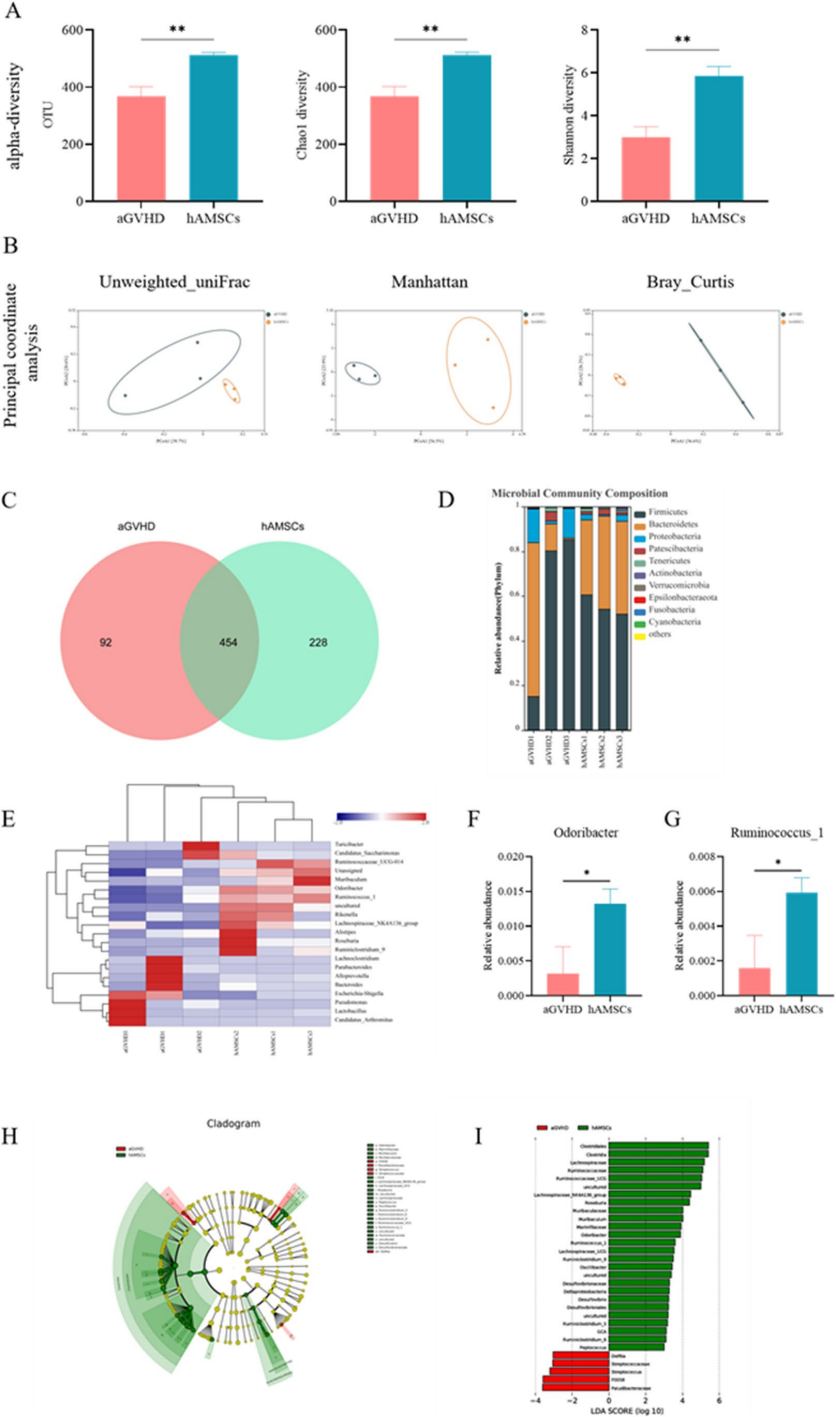
Gut microbiota alternations in aGVHD mice. (**A**) comparison of α-diversity. (**B**) comparison of β-diversity. (**C**) Venn diagram of OTUs. (**D**) Relative bacterial abundance at the phylum level. (**E**) Heatmap of species abundance at the genus level. (**F**) Relative abundances of Odoribacter at the genus level. (**G**) Relative abundances of Ruminococcus_1 at the genus level. (**H**) Cladogram based on LEfSe analysis. (**I**) LDA score computed from features differentially abundant between aGVHD and hAMSCs groups. (A-I) *n* = 3 mice per group. Values were presented as mean ± SD

The 16S rRNA sequencing was performed in fecal bacteria DNA isolated from different groups of mice. The α-diversity indexes, including OUT, Chao1 and Shannon indexes, manifested similar tendencies and hAMSCs treatment mice harbored a microbiota with significantly higher α-diversity relative to that of the aGVHD group (Fig. 6A). Principal coordinate analysis (PCoA) based on Unweighted-unifrac distance, Manhattan distance and Bray–curtis metric distance, yielded dispersed data points on the plots of the two groups, despite the difference was not significant (Fig. 6B). Subsequently, we assessed the landscape of the gut microbiota to further investigate the potential composition difference between the two groups. A total of 773 OTUs were calculated, and the hAMSCs group had higher OTUs compared with the aGVHD group (Fig. 6C). In terms of bacterial composition at the phylum level, *Firmicutes* and *Bacteroidetes* were the two most predominant phylum (Fig. 6D). We also organized a comparison heatmap for the analysis of gut microbiota between the two groups (Fig. 6E). The genus of *Odoribacter* and *Ruminococcus_1* displayed a relatively high abundance in the hAMSCs group, which are important microbes to maintain intestinal homeostasis (Fig. 6F, G). To confirm which bacterium was altered by hAMSCs treatment and in turn affected the disease progression against aGVHD, we performed high dimensional class comparisons using the linear discriminant analysis (LDA) of effect size (LEfSe) that detected marked differences in the predominance of bacterial communities between the two groups. As shown in (Fig. 6H, I), *Streptococcaceae* (the family and the genus *Streptococcus*), *Paludibacteraceae*, *F0058*, *Delftia* were the key types of bacteria contributing to gut microbiota dysbiosis in the aGVHD group. Nevertheless, the beneficial bacteria *Lachnospiraceae*, *Roseburia*, *Ruminococcaceae*, *Ruminiclostridium*, *Oscillibacter*, and *Clostridia* (the class and order *Clostridiales*) displayed a relative enrichment in the hAMSCs group, which might be associated with the hAMSCs-mediated alleviation of aGVHD. Collectively, the diversity and composition of gut microbiota were improved after hAMSCs administration and moved in an overall trend that was beneficial to the body.

### hAMSCs Regulated the Interactions between Gut Microbiota and Intestinal Immunity

**Fig. 7:**
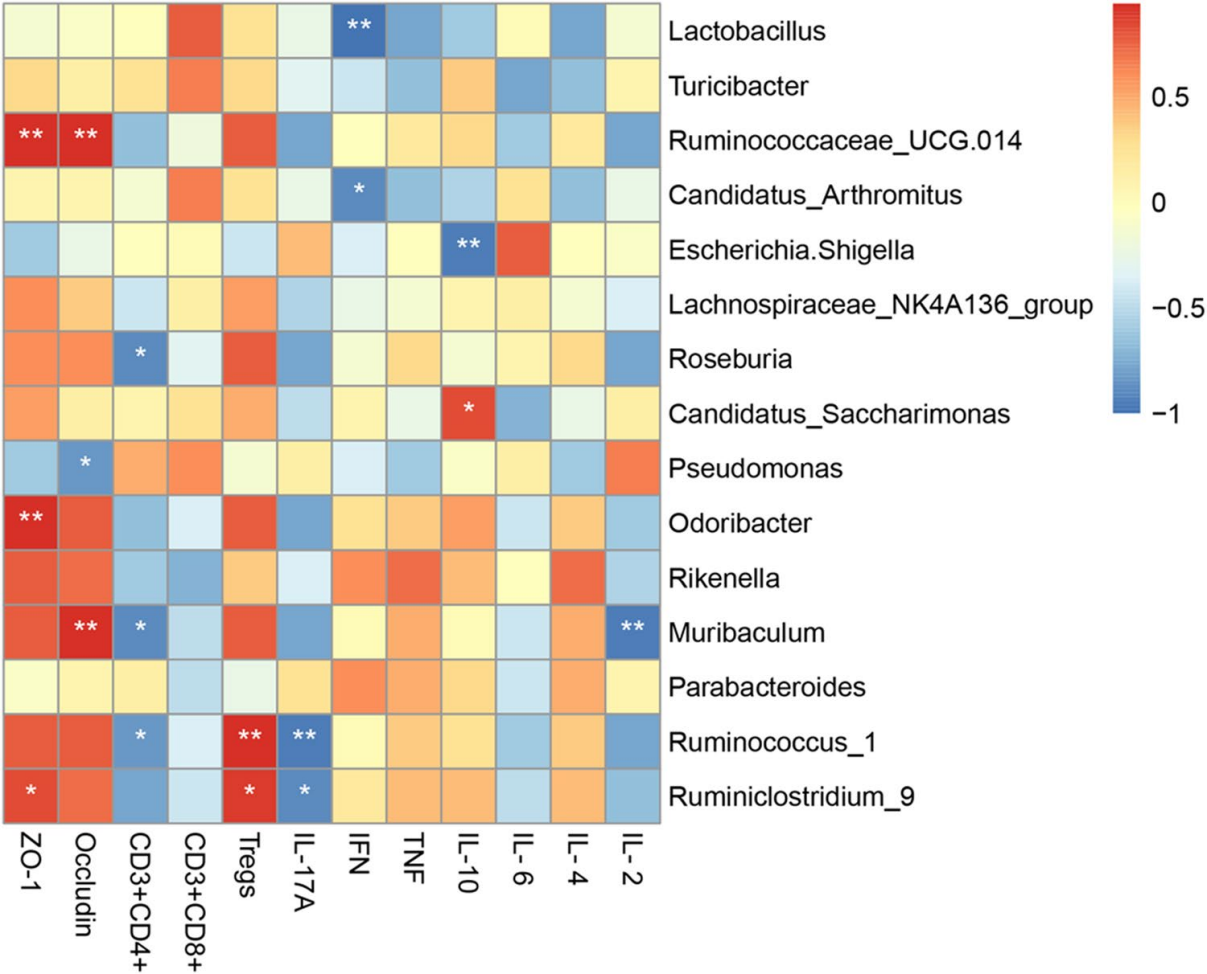
Correlation analysis between gut microbiota and intestinal immunity. Spearman’s correlation analysis was conducted between the relative abundance of 15 different gut microbiota (at the genus level) and the mRNA expression level of TJs, the percentage of immune cells as well as the concentration of cytokines in the intestines among the two groups. Spearman r values range from -0.5 (blue) to 0.5 (red)

To understand the potential relationship between the gut microbiota and intestinal mucosal barrier, the correlations between the TJs and gut microbiota at genus level were analyzed by Spearman’s correlation analysis. As shown in Fig. 7, there was a positive relation with ZO-1 and beneficial bacteria, *Ruminococcaceae_UCG.014*, *Muribaculum*, *Ruminococcus_1* and *Ruminiclostridium_9*. The relative abundance of the beneficial bacteria, *Roseburia*, *Odoribacter*, *Ruminococcus_1* and *Ruminococcaceae_UCG.014* showed dramatically positive correlations with Occludin. We next conducted a correlation analysis between gut microbiota and intestinal immune barrier by Spearman’s rank correlation method (Fig. 7). We observed that the increase of *Roseburia*, *Muribaculum* and *Ruminococcus _1*, was negatively correlated with the percentage of CD3 + CD4 + T cells. Additionally, the increase in beneficial bacteria, *Ruminococcus_1* and *Ruminiclostridium _9*, was positively correlated with the percentage of Tregs whereas negatively correlated with IL-17. The abundance of *Lactobacillus* and *Candidatus_Arthromitus* were negatively correlated with IFN-γ. There was a negative correlation between the abundance of *Muribaculum* and IL-2. The increase in *Candidatus_Saccharimonas* abundance and decrease in *Escherichia.Shigella* abundance were positively correlated with IL-10 level (Fig. 7). The results of correlation analysis indicated that hAMSCs improved the inflammatory environment of the host and regulate intestinal homeostasis which may be associated with the gut microbiota modulation, and consequently prevented aGVHD.

## Discussion

Advanced in microbial analysis have provided new insights into the complex interactions between the host and gut microbiota [18]. The gut microbiota could be altered after allo-HSCT and is closely associated with aGVHD, suggesting that gut microbiota may be a new target for aGVHD treatment [5]. Numerous studies including our recently study have explored and confirmed the therapeutical effect of MSCs on aGVHD [15, 19, 20]. However, the effect on gut microbiota during this period remains unclear. In this study, we investigated the impact of hAMSCs on aGVHD and potential mechanisms therein in vivo. Consistent with previous studies [15, 21–23], our results indicated that hAMSCs have favorable protective effect on aGVHD in mice, as evidenced by attenuated aGVHD symptoms and prolonged survival. We also demonstrated that hAMSCs displayed immunosuppressive effect through reducing donor T cell expansion while enhancing Tregs generation in aGVHD. Meanwhile, hAMSCs could downregulate pro-inflammatory cytokines and upregulate anti-inflammatory cytokines in vivo, so as to inhibit inflammatory responses.

The intestinal barrier, a dense structure composed of a monolayer of intestinal epithelial cells, maintains its integrity and prevents translocation of luminal microbiota in the healthy state [24]. During allo-HSCT, conditioning regiments damage the intestinal barrier, which is the initial step in the development of aGVHD, as it permits the translocation of bacteria across the barrier and leads to disrupting intestinal immune homeostasis [24, 25]. Accumulating evidence demonstrates that MSCs possess the capability to tissue repair, which were proven to repair the intestinal barrier integrity in colitis mice [26]. Tight junction proteins, including ZO-1 and Occludin, have an essential role in intestinal barrier function and translocation of the gut microbiota over the impaired intestinal barrier provokes inflammation [27]. D-LA is derived from intestinal bacteria, and DAO mainly concentrates in the intestinal mucosa. Thus, high concentration of plasma D-LA and DAO might partly reflect changes in intestinal permeability and intestinal barrier function [28]. In present, we found that the expression levels of TJs increased dramatically after hAMSCs administration, whereas the plasma levels of D-LA and DAO significantly decreased, indicating that hAMSCs could ameliorate intestinal barrier dysfunction and recovery the intestinal permeability.

The gut is one of the organs most severely affected by aGVHD and research has highlighted that bacterium, particularly the gut microbiota plays a central role in aGVHD pathogenesis and development [5, 6]. As reported in previous studies, the loss of bacterial diversity of allo-HSCT patients was associated with increased mortality from aGVHD [29, 30]. Fecal domination by opportunistic pathogens *Enterococcus* and *Proteobacteria* have also been linked to increased GVHD [6, 31]. For the past decades, increased attention has been paid to the correlation between the gut microbiota and MSCs treatment in inflammation diseases. MSCs have been proven to ameliorate gut microbiota dysbiosis of rheumatoid arthritis, sepsis and inflammatory bowel disease [32–34]. To further explore the mechanism by which hAMSCs ameliorate aGVHD, we investigated the impact of hAMSCs on the gut microbiota. In current study, hAMSCs administration showed a considerable effect on the α-diversity of the microbial communities compared with no transplantation, indicating that the abundance of gut microbiota could be changed after hAMSCs treatment. However, there was no significant difference in the β-diversity measurements, possibly because of the small sample sizes of both groups or the insufficient treatment cycle of hAMSCs. The analysis using comparison heatmap in genus levels indicated that hAMSCs administration increased the abundance of beneficial bacteria, *Odoribacter* and *Ruminococcus_1* in the intestinal flora, which were short-chain fatty acids (SCFAs) producers with anti-inflammatory properties [35, 36]. To identify the underlying dominant bacteria mediated through hAMSCs transplantation, LEfSe analysis was conducted between the two groups. Compared to aGVHD group, hAMSCs treatment significantly increased the abundance of some beneficial microbes related to anti-inflammatory effects, such as *Lachnospiraceae*, *Roseburia*, *Ruminococcaceae*, *Ruminiclostridium*, *Oscillibacter*, and *Clostridia*, which were all described to be beneficial for gut microbiota metabolite SCFAs production [37–42]. Therefore, our observations indicated that after hAMSCs administration, the gut microbiota dysbiosis was ameliorated, which is manifested by the improved diversity and the increase relative abundance of beneficial bacteria.

 aGVHD is a complicated inflammatory process that is initiated by the destruction of the intestinal barrier integrity follow up with the translocation of intestinal microbiota and their components. The recognition of damage-associated molecular patterns and pathogen-associated molecular patterns by antigen presenting cells induces pro-inflammatory response including activation of T cells and cytokine storm to aggravate intestinal barrier damage and promote the development of aGVHD [24, 43]. On the other hand, the gut microbiota and microbiota-derived metabolites such as SCFAs play important roles in the maintaining of intestinal barrier integrity and intestinal homeostasis as well as shaping the mucosal immune system, balancing host defense with microbial components and metabolites [24, 44]. Intercommunication between the host and gut microbiota at the intestinal barrier regulate mucosal and systemic immune responses and, in pathological states, may result in aGVHD [6, 45, 46]. It suggested that the gut microbiota is closely related to the expression of tight junction proteins [47]. The present study showed that the mRNA expression of TJs ZO-1 and Occludin was negatively correlative to the relative abundance of harmful bacteria whereas had a positive correlation to beneficial bacteria, indicated that the gut microbiota was important to maintain the integrity of the intestinal barrier. Additionally, the gut microbiota is also very important in maintaining the intestinal immune barrier [47]. We observed that some of the beneficial bacteria were negatively correlated with the percentage of CD3 + CD4 + T cells, but positively correlated with the percentage of Tregs. Here, we revealed that beneficial bacteria, mostly a SCFAs producer, were negative correlated to pro-inflammatory cytokines but positive correlated with anti-inflammatory cytokines, while harmful bacteria are conversely correlated. In summary, the significant differential gut microbiota between treatments suggested a full picture of the interactions between the gut microbiota and the intestinal barrier, immunity.

There were still several limitations worth discussing in our experiments. Firstly, although our study explored the effects of hAMSCs on immunity, intestinal barrier and gut microbiota in aGVHD, the underlying mechanism still needs to be verified in the further study. Secondly, gut microbiota depletion and fecal microbiota transplantation are needed to address the causal correlations among the gut microbiota, intestinal barrier and immunity. Thirdly, further research investigating the impact of hAMSCs on microbiota-derived SCFAs and other metabolites is warranted.

In conclusion, our research suggested that hAMSCs alleviated aGVHD by promoting gut microbiota normalization and regulating the interactions between the gut microbiota and intestinal barrier, immunity. Our study is the first systematically to define the effects and underlying mechanisms of hAMSCs regulating the gut microbiota and intestinal immunity in aGVHD.

## Data Availability

The datasets generated during and/or analyzed during the current study are available from the corresponding author on reasonable request.
